# Challenging measles case definition: three measles outbreaks in three Health Regions of Portugal, February to April 2018

**DOI:** 10.2807/1560-7917.ES.2018.23.28.1800328

**Published:** 2018-07-12

**Authors:** Gonçalo Figueiredo Augusto, Diogo Cruz, Andreia Silva, Natália Pereira, Bárbara Aguiar, Ana Leça, Elisabete Serrada, Paula Valente, Teresa Fernandes, Fernando Guerra, Paula Palminha, Elsa Vinagre, Sílvia Lopo, Rita Cordeiro, Emma Sáez-López, Maria Neto, Paulo Jorge Nogueira, Graça Freitas

**Affiliations:** 1 Directorate-General of Health, Lisbon, Portugal; 2 National Institute of Health Dr Ricardo Jorge, Lisbon, Portugal; 3 European Public Health Microbiology Training (EUPHEM), European Centre for Disease Prevention and Control (ECDC), Stockholm, Sweden; 4 North Region Health Administration (SNS), Porto, Portugal

**Keywords:** measles, outbreak, Portugal, Genotype B3, Healthcare workers

## Abstract

We report three simultaneous measles outbreaks with 112 confirmed cases in three Health Regions of Portugal, from February to April 2018. The mean age of cases was 30 years, 79% worked in a healthcare setting and 87% were vaccinated. Genotype B3 was identified in 84 cases from the three outbreaks. Primary cases in each outbreak were imported. Several cases presented with modified measles, highlighting the importance of rethinking the measles case definition for vaccinated cases.

We present preliminary findings and implemented control measures of three simultaneous measles outbreaks that occurred in Portugal between February and April 2018. One of the outbreaks took place in a hospital and represented a particular challenge for epidemiological and laboratory investigations as a substantial number of vaccinated healthcare workers (HCWs) developed benign clinical signs and symptoms of measles. We discuss these findings and highlight the need to expand the European Union (EU) measles case definition, in order to increase sensitivity in case capture among vaccinated individuals with modified measles and who do not meet the current European Union (EU) case definition.

## Case definition

Measles case definition used for epidemiological surveillance in Portugal is based on the EU case definition [1]. A possible case is any person who meets clinical criteria (i.e. fever, maculopapular rash, and any of cough/coryza/conjunctivitis); a probable case is any person who meets clinical criteria and has an epidemiological link to a confirmed case; a confirmed case is any possible case with laboratory evidence of infection with measles wild virus (i.e. detection of viral RNA in a biological sample and/or a positive IgM result in serum), determined by the World Health Organization (WHO)-certified national reference laboratory for measles and rubella National Institute of Health – Instituto Nacional de Saúde Doutor Ricardo Jorge, Lisbon [2]. Cases are discarded when clinical, epidemiological or laboratory criteria are not met, taking into account vaccination history and risk of measles infection in the community or abroad, following WHO criteria [3].

However, symptoms in modified measles cases are masked meaning that cases do not present with the usual signs and symptoms of classic measles, this making a clinical diagnosis more challenging. Modified measles mainly affects young adults who have been vaccinated, suggesting that they could have suboptimal protection against measles whether it be from insufficient number of vaccination doses or that the immunity to disease has waned over time as revealed in the National Serological Survey (2015/2016) [4]. Therefore, the case definition used during this outbreak was expanded to increase sensitivity: clinical criteria included any person with a maculopapular rash, or fever or any of the following three symptoms: cough, coryza, conjunctivitis. Epidemiological criteria included any person with a link to the hospital or with a confirmed measles case.

## Outbreak description

On 9 March 2018, a laboratory-confirmed measles case was notified by INSA. It corresponded to an unvaccinated French citizen, recently arrived in the North Health region from the Aquitaine Region, where a measles outbreak has been ongoing [5]. Following the laboratory notification, the case was clinically notified on 12 March in the National System for Epidemiological Surveillance (Sistema Nacional de Vigilância Epidemiológica, SINAVE), which is an integrated clinical and laboratory electronic system of mandatory notification. This case was the source of infection for three additional cases in close relatives that either lived with or visited the case; all had been vaccinated with two measles-mumps-rubella (MMR) doses. No further cases were related to this chain of transmission.

On 13 March 2018, the clinical director of a hospital in Oporto reported 24 suspected measles cases among the hospital’s HCWs to public health authorities. All suspected cases had a link with the Emergency Department and presented with maculopapular rash, tachycardia, low fever and headache. The following day, INSA confirmed the first two cases along with a third, who was not a HCW and was admitted to another hospital in the city. Epidemiological investigations led to the retrospective identification of the imported primary case, who was an unvaccinated individual from Italy who arrived in Oporto 10 days before the symptom onset and who went to the Emergency Department when they developed a rash. Overall, there were 103 confirmed measles cases associated with this primary case. Most cases were HCWs (n = 87; 84.5%), of which 10 (11.5%) were vaccinated with one dose of a measles-containing vaccine, 66 (75.9%) with two doses, four (4.6%) with three doses, and seven (8.0%) were unvaccinated.

On 26 and 28 March, two cases with history of recent travel to two different African countries and both having a stopover at the same airport on the same day during the incubation period (and evidence of remaining in the same waiting room) were notified in SINAVE. One of the cases was vaccinated with two MMR doses and did not infect further cases. The other case was unvaccinated and infected three additional cases, one at work and two contacts in a hospital Emergency Department.

**Figure 1:**
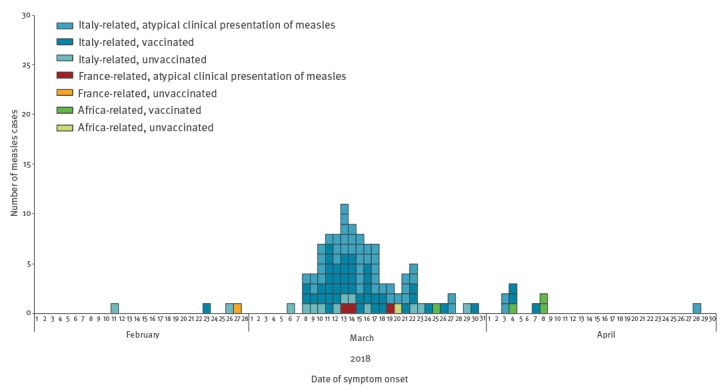
Confirmed measles cases by date of symptom onset, chain of transmission and vaccination status, Portugal, 11 February–28 April 2018 (n = 112)

Source: Direção-Geral da Saúde, Sistema Nacional de Vigilância Epidemiológica.

From 12 March until 31 May, a total of 440 suspected measles cases were notified in SINAVE, of which 112 (25.5%) were laboratory-confirmed in INSA, 303 (68.9%) were discarded and 25 (5.7%) were still under investigation. Figure 1 shows the distribution of all confirmed measles cases by date of symptom onset and chain of transmission. Overall, 47 (45.6%) cases in this chain of transmission had benign clinical signs and symptoms of measles. History of vaccination was verified from individuals’ medical charts or from the national immunisation registry.

Of the 112 confirmed measles cases the mean age was 30 years (SD: 7.7) and 65 cases were female. Preliminary findings show that 111 confirmed measles cases occurred in adults (≥ 18 years), with an age range of 20–54 years and one case in a 3-year-old child vaccinated with one MMR dose (Table 1). Among the 112 confirmed cases, 83 (74.1%) were vaccinated with two or more doses of a measles-containing vaccine. Fifty (44.6%) confirmed cases did not meet the clinical criteria from the EU case definition; among them, 24 of 50 had a maculopapular rash and fever as clinical presentation and 13 cases (11.6%) only had a maculopapular rash. Twenty-one cases (18.8%) were confirmed through laboratory results of second samples, where an increase of either IgM, IgG or both was verified (Table 1). Among the 88 cases where viral RNA was detected, 84 cases could be genotyped. Genotype B3 was identified in cases from all the three chains of transmission, although the four cases from the Africa-related chain of transmission had a 5 nucleotide difference from the genotype B3 identified in the other two chains of transmission, which was phylogenetically indistinct.

**Table 1:** Characteristics of measles cases by chain of transmission Portugal, 11 February − 28 April 2018 (n = 112)

	France-related chain of transmission	Italy-related chain of transmission	Africa-related chain of transmission	Total	
	n	n	n	n	%
**Total**	**4**	**103**	**5**	**112**	**100.0**
**Sex**					
Female	2	62	1	**65**	**58.0**
Male	2	41	4	**47**	**42.0**
**Age group (years)**					
< 1	0	0	0	**0**	**0.0**
1–9	0	1	0	**1**	**0.9**
10–19	0	0	0	**0**	**0.0**
20–29	2	52	1	**55**	**49.1**
30–39	2	40	3	**45**	**40.2**
40–49	0	8	1	**9**	**8.0**
50–59	0	2	0	**2**	**1.8**
≥ 60	0	0	0	**0**	**0.0**
**Vaccination status**					
Not vaccinated	1	13	1	**15**	**13.4**
1 dose	0	12	2	**14**	**12.5**
2 doses	3	74	2	**79**	**70.5**
3 doses	0	4	0	**4**	**3.6**
**Occupation**					
Non-Healthcare workers	3	16	4	**23**	**20.5**
Doctors	1	33	0	**34**	**30.4**
Nurses	0	20	0	**20**	**17.9**
Allied professionals	0	15	1	**16**	**14.3**
Medical/Nursing students	0	18	0	**18**	**16.0**
Other Healthcare workers	0	1	0	**1**	**0.9**
**Measles symptoms**					
Maculopapular rash + Fever + Cough/Coryza/Conjunctivitis	1	56	5	**62**	**55.4**
Maculopapular rash only	2	11	0	**13**	**11.6**
Fever only	0	2	0	**2**	**1.8**
Maculopapular rash + Fever	1	23	0	**24**	**21.4**
Maculopapular rash + Cough	0	1	0	**1**	**0.9**
Maculopapular rash + Coryza	0	7	0	**7**	**6.2**
Fever + Coryza	0	1	0	**1**	**0.9**
Fever + Cough + Coryza	0	1	0	**1**	**0.9**
Cough + Coryza	0	1	0	**1**	**0.9**
**Laboratory results**					
Detection of viral RNA	3	80	5	**88**	**78.6**
Positive IgM	0	3	0	**3**	**2.7**
Increase of both IgM and IgG in a pair of samples	1	7	0	**8**	**7.1**
Increase of IgM in a pair of samples	0	3	0	**3**	**2.7**
Increase of IgG in a pair of samples	0	10	0	**10**	**8.9**
**Genotype**					
B3	2	78	4	**84**	**64.1**

Source: Direção-Geral da Saúde, Sistema Nacional de Vigilância Epidemiológica, Instituto Nacional de Saúde Dr Ricardo Jorge.

**Figure 2:**
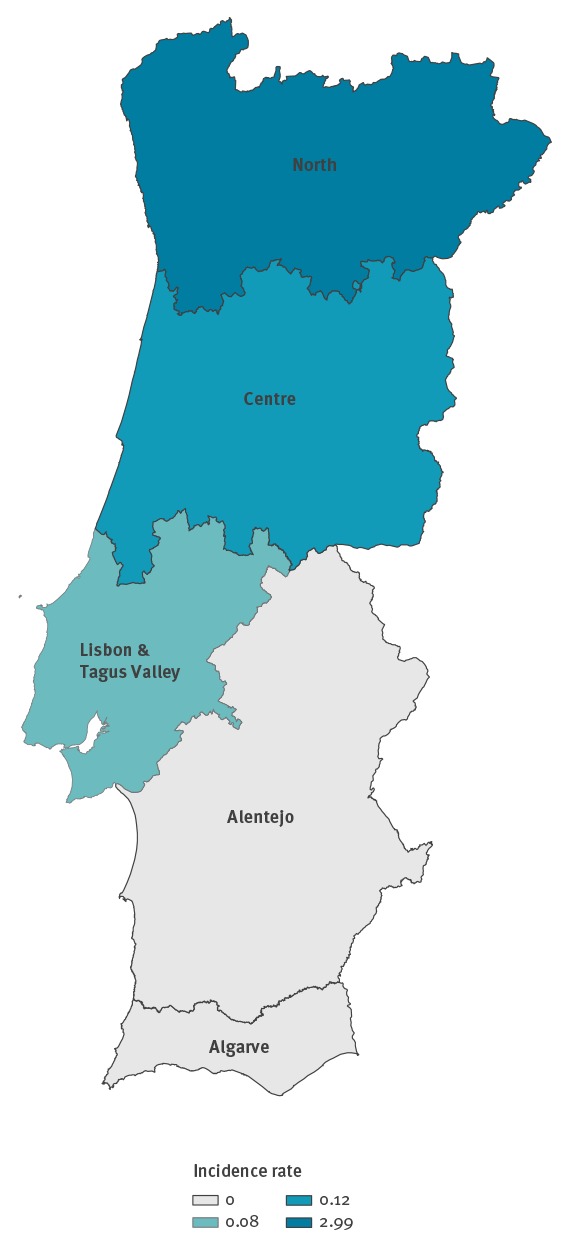
Incidence rate of confirmed measles cases per 100,000 population by health region, Portugal, 11 February−28 April 2018 (n = 112)

Source: Direção-Geral da Saúde, Sistema Nacional de Vigilância Epidemiológica. *Note: Map developed by GeoSaúde (*http://www.geosaude.dgs.pt/*).*

The measles outbreaks affected three of the seven Portuguese Health Regions (Figure 2), with the majority of cases 107 of 112 (95.5%) reported in the North Health Region.

## Control measures

Following the laboratory notification of the first confirmed measles case, DGS issued a warning to healthcare services that was followed by recommendations and guidelines regarding diagnosis, early detection and response to measles cases, within the scope of the National Measles Elimination Programme [6].

In order to control the outbreak on a local level and mitigate transmission outside the healthcare setting, an Emergency Response Team comprising of hospital and public health professionals was created in the affected hospital in Oporto [7]. A vaccination point within the hospital was set-up allowing for rapid vaccination of close contacts and unvaccinated individuals.

During the outbreaks, all suspected measles cases reported were investigated and control measures were promptly implemented to contain further transmission. Local public health teams undertook extensive contact tracing for all suspected measles cases. Surveillance and control measures included immediate isolation of suspected cases, verification of immunisation status of close contacts and administration of prophylactic immunoglobulin or MMR vaccine, whenever necessary. In addition, control measures were complemented with broader public health measures, including the dissemination of key documents to support prevention and control measures [8,9] and raising public awareness about the importance of vaccination through numerous reports in the national media as well as a large media campaign. Daily press releases and epidemiological bulletins were issued by DGS while the outbreaks lasted.

As the primary or index cases did not originate in Portugal or had stayed in another country during their incubation period, the director-general of Health in Portugal notified the Health Authorities from these countries regarding the cases, following the International Health Regulations [10].

## Discussion

Following 12 years without endemic measles transmission, Portugal experienced two measles outbreaks in 2017 [11] and, so far, three measles outbreaks in 2018. In two of these transmission mainly occurred in the community setting, whereas one mainly occurred in a healthcare setting. The high coverage of measles vaccination and the timely implementation of control measures allowed for the rapid containment of measles and interruption of all chains of transmission. The outbreaks were declared over on 10 June 2018 and since 29 April 2018 no new cases have been detected [12]. The Immediate isolation of cases, extensive contact tracing and vaccination were crucial to contain the outbreak in the Oporto hospital and avoid its spread to the community.

Vaccination or acquired immunity after illness constitute adequate protection against measles [13]. Since the measles vaccine was introduced in the Portuguese National Immunisation Programme in 1974, the country has achieved a consistent and sustained high immunisation coverage against measles (> 95%) [11,14].

HCWs are at higher risk of measles exposure because the high intensity of the exposure and subsequent transmission to vulnerable patients [15]. According to the National Measles Elimination Programme, HCWs are recommended to receive two doses of measles vaccine (either single measles-containing vaccine or MMR) or to have evidence of previous measles infection [6]. However, measles outbreaks in healthcare settings are becoming more frequent in the European Region [15-18]. Countries, such as Portugal, which maintained a high vaccination coverage for many years and had eliminated measles, are at greater risk of modified measles cases emerging during outbreaks. This is due to suboptimal protection against measles, either from insufficient number of vaccine doses or waning immunity from the vaccine over time (as indicated by the National Serological Survey 2015/2016). Modified measles mainly affect young adults who were adequately vaccinated but with the last dose of the vaccine administered more than 10 years prior.

In one chain of transmission, a hospital cluster was identified and most cases were HCWs vaccinated with two or more doses of MMR vaccine. This was described in other outbreaks [18] and may be related to waning of vaccine-induced immunity in the absence of natural boosting by the wildtype virus [19].

Modified measles cases has been described in vaccinated individuals [20,21]. In the outbreaks reported here, this was the case in 50 of 112 (44.6%) confirmed cases. Early findings of modified measles led us to expand the case definition initially in place in order to increase sensitivity. Interestingly, 5 of 112 (4.5%) confirmed cases did not have a maculopapular rash, and their symptoms would have been easily mistaken for other clinical conditions if they were not investigated in the context of a measles outbreak. Also, laboratory confirmation was only possible due to the collection of second serum samples in 21 of 112 cases, where an increase of IgM or IgG antibodies was verified [22].

The outbreaks described here, which included a number of cases with modified measles and a large number of cases among vaccinated HCWs, highlight the need for further investigation in order to recommend innovative approaches in future outbreaks: Nearly half of these cases would not have been identified using the current EU case definition. Thus, in light of these new findings and in order to increase sensitivity in case capture in the context of an outbreak, it would be important to develop an additional case classification suited for a community with high vaccination coverage with epidemiological criteria, that may lead to the definition of risk levels for public health intervention according to the type of exposure or depending to exposure to cases of reinfection.
